# Lyssin in Hydrophobia; Euthanasia from the Simillimum

**Published:** 1881-07

**Authors:** E. W. Berridge

**Affiliations:** London


### A CASE OF HYDROPHOBIA.

### Euthanasia obtained by the Corresponding Nosode.

E. W. Berridge, M. D., London.

"A Fourth mode of employing medicines in diseases has been attempted to be created by means of Isopathy, as it is called; that is to say, a method of curing a given disease by the same contagious principle that produces it. But even granting this could be done, WHICH WOULD CERTAINLY BE A MOST VALUABLE DISCOVERY, yet, after all, seeing that the miasm is given to the patient highly dynamised, and thereby consequently, to a certain degree, in an altered condition, *the cure is effected only by opposing a simillimum to a simillimum.*"

A boy, aged 15. In 1874, had abscesses in hip and sacrum from diseased bone. I cured him of this, and he remained well for two years. On November 5th, 1877, I prescribed *Sulphur* for rheumatic pains. On December 5th, 1877, he was bitten by a dog on right arm and left leg. The same day I gave him one dose of *Hydrophobinum* c. m. (Swan). The wounded parts had been neither excised nor cauterized. All that I could learn about the dog, was that it ran into the shop, the boy shouted at it to drive it out, upon which it flew at him and bit him; the boy was then rescued by his father, and the dog walked quietly out of the shop, ran down the street, and bit another dog, which was ill for a week or two afterwards. On October 31st, 1878, I prescribed *Lycop.* for rheumatic pains, and on November 14th, *Syphilinum.* On November 26th, he was better, and I did not see him again until his present illness, November 11th, 1879.

About six or eight weeks before the fatal attack, he had tingling in the bitten wrist, where the scar was still visible, but it ceased after twenty-four hours.

On November 6th, these symptoms returned—a tingling, as from an electric battery, in the wrist.

On November 7th, the symptoms increased. Pressure on the bitten wrist threw out a feeling of soreness all up arm, with a burning sensation; pain like rheumatism extending to shoulder; tingling in wrist as before.

November 8th.—Dull aching pain in shoulder increased gradually, and by 5 P. M., he could not use the arm from pain. The tingling and soreness continued, irrespective of pressure on the bite, though this made them worse and caused tingling or prickling like pins and needles all up arm. The whole nervous system seemed affected by the tingling, until the vibrations became so great that his parents could feel the quivering as if he were charged with electricity. He felt sleepy and retired, but was very restless at night and did not sleep. Through the night and next day the pain in the shoulder increased.

November 9th.—The aching continued, extending up neck and chest; relieved by the application of hot salt bags; there was also tighthess of chest, and he could not get his breath. About 8 P. M. he first refused water. His parents were going out for a walk, and he asked them not to be away long, as he felt he was going mad. On their return, he said he could not drink. He then tried to drink, but as the glass approached his lips he started, and said that he felt a freshness proceed from the water which made him shudder. From that time he drank nothing, and since his dinner, on November 8th (when he only ate a little), has eaten scarcely any solid food. During the night he became much worse, with craving for drink, and pain extending from shoulder to muscles of chest and throat. His father had diagnosed his case as rheumatism, and gave him *Acon.* and *Bry.*

November 10th. His father sent for a neighboring physician, who first gave him, at 10 A. M., a brown bitter powder, and afterwards *Bell.* and *Lach.* in low dilutions. What the "brown bitter powder" was, I could not ascertain; the physician told me nothing about it, but I learnt it afterwards from the father. On this day, he had frequent paroxysms, during which he would stand up on the bed, jump and shriek. When he felt one coming on, he would exclaim, "Hold me tight, I shall hurt you or myself." The paroxysms gradually increased till I saw him, occurring every two or three minutes for an hour; then there was an interval of half an hour. Tongue and throat parched; great burning heat in throat; froth in mouth which he could not eject.

November 11th. I was called in consultation in the early morning. His father says that nothing has relieved him, but that he became worse and worse, especially since 4 or 5 A. M., till I prescribed for him, after which there was an immediate improvement. When I first saw him, he was in a violent paroxysm, crying out that he wanted water, and none had been given him for a long time, and reproaching his parents for their cruelty in withholding it; then, when they offered it to him, he told them to take it away else he should bite the spoon in two; shrieked, jumped about, threw himself about wildly, being held with difficulty, and threatened those around him. The *slightest* draught of air made him shudder and scream. He says he can feel a cool emanation from the water when near him. Pulse 150, feeble. Tongue covered with foam. Says that the touch of a cold hand, or the entrance of cold air into the mouth, "sends an electric battery through him." If he tries to drink, he gasps and shudders. I gave him a spoonful of water; he took it with a sudden snap and gulp, and was then convulsed, jumped about, and seized hold of his father. Yesterday he could lie quietly for an hour unless disturbed, or unless he tried to drink; subsequently, he was unable to lie, but had to sit up; for the last hour or two, he has been obliged to stand. Since 4 or 5 A. M. the paroxysms have come on every fifteen minutes, and are decidedly more severe. He says he cannot get a full breath from a feeling of a ton weight on chest. Urine scanty. Since the 9th he has had no sleep; has not been able to swallow liquids, and has only eaten a sponge-cake and a few grapes; he has excessive thirst, and there is froth before his mouth.

At 7.8 A. M., just after a very severe paroxysm, I gave him a dose of *Hydrophobinum* c. m. (Swan). In a few minutes he sat down quietly, which he had not done for some time. At 7.18 A. M. a dose. He takes the globules with a hurried gulp. He now seemed quieter, repeated a prayer after his father and asked his forgiveness for what he had said to him during the former paroxysms. He was quite rational and conscious. He thinks he will die, and says he is quite ready, telling his relations not to grieve. At 7.30 A. M. I gave a dose, which he took more quietly. The other physician says that this is the best interval he has had since 3 or 4 A. M. At 7.45 A. M. a dose; has been walking about the room supported by his parents; unnaturally talkative, but rational. At 8 A. M. a dose; says head feels clearer. At 8.7 A. M., took a teaspoonful of warm tea better than he took the last water. At 8.15 A. M. a dose. 8.22 A. M. *Has had no paroxysm since the first dose,* except a little shuddering from a draught and when drinking the tea; but he now jumped up with momentary paroxysm of gasping. He says his breath has been shorter since the last dose, and thinks he has had too much. At 8.38 A. M. the paroxysms increased, but were momentary; he jumps up gasping, the last time with a shriek, as when I first saw him. Previous to this, he had been quieter for some time. Repeated the dose. For the first time since I saw him has had a little saliva in mouth, but he cannot swallow it. *Has not craved for drinks since the first dose.* At 8.45 A. M. jumped up with a shriek, a momentary paroxysm, without perceptible cause. At 8.47 A. M., two more paroxysms. Gave another dose; directly afterwards had a still more severe attack, though less severe than the first I witnessed. By 9.05 A. M. had had twenty repeated attacks every minute or half minute, but lasting only about thirty seconds. Pressing him tightly at hypochondria helps him to get his breath. He thinks he is dying. 9.12 A. M., has been better for the last five minutes; spits more. I was now obliged to leave him. After the first attack in which I saw him had passed off, while quietly walking about the room with his parents, he said he felt strong enough to take us all up and shake us. During the severe paroxysm which occurred just before I left him, he assured us that he would not hurt us, and asked us to strike him so as to make him call out, as this enabled him to get his breath.

His father furnished me with the following report of the boy's symptoms after I left him. The spitting and vomiting of phlegm and froth continued for several hours. *He could not spit before I gave him the medicine;* but the ability to do so came on a little just before I left him, and was fully established immediately afterwards. *No thirst or asking for drink after the first dose till just before death.* The paroxysms changed in character, consisting only of catching of breath, relieved by any one jerking the hypochondria forcibly inwards and by beating the abdomen. He would ask them to strike him hard, it did not hurt him; he wished to be struck *suddenly* when the attack came on. This oocurred every few minutes till 2 P. M. From 2 P. M. till 4 P. M. rapid vomiting of liquid, at first froth and phlegm, then dark brown liquid. After 4 P. M. he stood stooping, with his hands on his knees; he said that if he raised himself up, he should die at once. This lasted till 9 P. M., vomiting all the time. About 8 P. M., there was fæcal vomiting. At 5 or 6 P. M. there was involuntary urination. After 2 P. M. the catching of breath

22

gradually became more feeble. *No pain after* 2 *P. M.* The vomiting lasted till he died, at 9 P. M. At 8.30 P. M. he ate four sponge-cakes dipped in sherry and water, and sat up, saying that he felt refreshed by them; but he soon vomited them, partly through the nostrils, and died. An hour before death was quite conscious; just before there was a little wandering. He took four or five doses after I left him.

This case demonstrates that in incurable cases, even of the most painful character, the administration of the homœopathic remedy is all-sufficient to procure euthanasia.

